# Strong correlation between the rates of intrinsically antibiotic-resistant species and the rates of acquired resistance in Gram-negative species causing bacteraemia, EU/EEA, 2016

**DOI:** 10.2807/1560-7917.ES.2019.24.33.1800538

**Published:** 2019-08-15

**Authors:** Vincent Jarlier, Liselotte Diaz Högberg, Ole E Heuer, José Campos, Tim Eckmanns, Christian G Giske, Hajo Grundmann, Alan P Johnson, Gunnar Kahlmeter, Jos Monen, Annalisa Pantosti, Gian Maria Rossolini, Nienke van de Sande-Bruinsma, Alkiviadis Vatopoulos, Dorota Żabicka, Helena Žemličková, Dominique L Monnet, Gunnar Skov Simonsen, EARS-Net participants

**Affiliations:** 1 Sorbonne Universités (Paris 06) Inserm Centre d'Immunologie et des Maladies Infectieuses (CIMI), UMR 1135, Paris, France; 2 Assistance Publique – Hôpitaux de Paris, Pitié-Salpêtrière hospital, Laboratoire de Bactériologie-Hygiène, Paris, France; 3 European Centre for Disease Prevention and Control, Solna, Sweden; 4 Reference and Research Laboratory on Antimicrobial Resistance, Centro Nacional de Microbiología, Instituto de Salud Carlos III, Madrid, Spain; 5 Robert Koch Institute, Department for Infectious Disease Epidemiology, Berlin, Germany; 6 Department of Laboratory Medicine, Karolinska Institute, Stockholm, Sweden; 7 Department of Clinical Microbiology, Karolinska University Hospital, Stockholm, Sweden; 8 Medical Center – University of Freiburg, Department for Infection Prevention and Hospital Epidemiology, Freiburg, Germany; 9 National Infection Service, Public Health England, London, United Kingdom; 10 Clinical Microbiology, Central Hospital, Växjö, Sweden; 11 National Institute for Public Health and the Environment, Bilthoven, the Netherlands; 12 Department of Infectious Diseases, Istituto Superiore di Sanità, Rome, Italy; 13 Department of Experimental and Clinical Medicine, University of Florence, Italy; 14 Microbiology and Virology Unit, Florence Careggi University Hospital, Florence, Italy; 15 Pan American Health Organization/World Health Organization (PAHO/ WHO), Washington DC, United States; 16 Department of Public Health Policy, School of Public Health, University of West Attica, Athens, Greece; 17 Department of Epidemiology and Clinical Microbiology, National Medicines Institute, Warsaw, Poland; 18 National Institute of Public Health, National Reference Laboratory for Antibiotics, Prague, Czech Republic; 19 Department of Clinical Microbiology, Faculty of Medicine and University Hospital, Charles University, Hradec Kralove, Czech Republic; 20 Department of Microbiology and Infection Control, University Hospital of North Norway, Tromsø, Norway; 21 Research Group for Host-Microbe Interaction, Faculty of Health Sciences, UiT – The Arctic University of Norway, Tromsø, Norway; 22 The members of the group are listed at the end of the article

**Keywords:** antibiotic resistance, bloodstream infection, Gram-negative bacilli, bacterial infections, antimicrobial resistance

## Abstract

**Background:**

Antibiotic resistance, either intrinsic or acquired, is a major obstacle for treating bacterial infections.

**Aim:**

Our objective was to compare the country-specific species distribution of the four Gram-negative species *Escherichia coli*, *Klebsiella pneumoniae, Pseudomonas aeruginosa* and *Acinetobacter* species and the proportions of selected acquired resistance traits within these species.

**Method:**

We used data reported for 2016 to the European Antimicrobial Resistance Surveillance Network (EARS-Net) by 30 countries in the European Union and European Economic Area.

**Results:**

The country-specific species distribution varied considerably. While *E. coli* accounted for 31.9% to 81.0% (median: 69.0%) of all reported isolates, the two most common intrinsically resistant species *P. aeruginosa* and *Acinetobacter* spp. combined (PSEACI) accounted for 5.5% to 39.2% of isolates (median: 10.1%). Similarly, large national differences were noted for the percentages of acquired non-susceptibility to third-generation cephalosporins, carbapenems and fluoroquinolones. There was a strong positive rank correlation between the country-specific percentages of PSEACI and the percentages of non-susceptibility to the above antibiotics in all four species (rho > 0.75 for 10 of the 11 pairs of variables tested).

**Conclusion:**

Countries with the highest proportion of *P. aeruginosa* and *Acinetobacter* spp. were also those where the rates of acquired non-susceptibility in all four studied species were highest. The differences are probably related to national differences in antibiotic consumption and infection prevention and control routines.

## Introduction

Bloodstream infections constitute a major disease burden in Europe. Gram-negative bacteria such as *Escherichia coli*, *Klebsiella pneumoniae*, *Pseudomonas aeruginosa* and *Acinetobacter* species are the most common organisms involved in these infections. Data from the European Antimicrobial Resistance Surveillance Network (EARS-Net) show that the proportional contribution of each bacterial species to the total number of blood isolates varies considerably between countries [1]. In particular *Acinetobacter* spp. and *P. aeruginosa* seem to be proportionally far more commonly isolated in some European countries than in others. Several studies have previously made the same observation [2-4].

Antibiotic resistance is a major obstacle for treating these serious infections. Resistance can be both intrinsic, i.e. due to a species’ innate ability to resist a particular antibiotic because of structural or functional characteristics, or acquired though a range of resistance mechanisms emerging either by mutation or acquisition of novel genes.

Among Gram-negative bacteria, intrinsic resistance varies markedly between species. For example, even wild-type isolates of *K. pneumoniae* are intrinsically resistant to penicillins whereas wild-type isolates of *E. coli* are susceptible to the same antibiotics. More importantly, every wild-type isolate of *Acinetobacter* spp. and *P. aeruginosa* is intrinsically resistant to numerous groups of antibiotics (e.g. aminopenicillin-β-lactamase inhibitor combinations, first- and second-generation cephalosporins, cefotaxime/ceftriaxone, ertapenem, trimethoprim, tetracyclines and older quinolones) [5], immediately excluding these as possible treatment alternatives. Acquisition of additional resistance traits can further reduce available treatment options, jeopardise the use of major remaining groups of antibiotics including β-lactams, fluoroquinolones and aminoglycosides. Bacteria can acquire multiple resistance mechanisms, leading to multidrug-resistant (MDR), extensively drug-resistant (XDR) or even pandrug-resistant (PDR) isolates [6]. Thus the overall pattern of resistance as presented in the laboratory reports reflects the intrinsic resistance characteristics of the species combined with any additional resistance trait acquired by the isolate.

Similar to the differences in the distribution of Gram-negative species between European countries, EARS-Net has also documented large differences in the percentage of acquired antibiotic resistance [1,2]. However, there is no study addressing the possible link between the ranking of the various species of Gram-negative bacteria and the percentage of acquired resistances in these species. As an example, most publications on resistance in *P. aeruginosa* and *Acinetobacter* spp. only focus on acquired resistance mechanisms or on clonal expansion of resistant epidemic clones, and a few others describe the intrinsic resistance characteristics of these species [7-10]. Correlation between intrinsic and acquired resistance is mentioned only from a mechanistic perspective, such as the link between inducible and de-repressed production of chromosomal AmpC β-lactamase [8], but not from a statistical or epidemiological point of view.

The objective of the present study was to assess the association between the proportion of the two most common intrinsically resistant species (*P. aeruginosa* and *Acinetobacter* spp.) among the four major Gram-negative species (*E. coli*, *K. pneumoniae*, *P. aeruginosa* and *Acinetobacter* spp.) and the percentage of selective acquired resistance traits in these species. As the data source, we used data reported to EARS-Net for countries in the European Union (EU) and European Economic Area (EEA) in the year 2016.

## Methods

### Data source and inclusion criteria

EARS-Net is a surveillance network which collects and analyses data from routine antibiotic susceptibility testing (AST) of bacterial pathogens from all 28 EU countries (Austria, Belgium, Bulgaria, Croatia, Cyprus, the Czech Republic, Denmark, Estonia, Finland, France, Germany, Greece, Hungary, Ireland, Italy, Latvia, Lithuania, Luxembourg, Malta, the Netherlands, Poland, Portugal, Romania, Slovakia, Slovenia, Spain, Sweden and the United Kingdom) and two EEA countries (Iceland and Norway). The network is coordinated by the European Centre for Disease Prevention and Control (ECDC). Only AST results for selected important antibiotics active against invasive bacterial infections are included in EARS-Net. The AST results are ascertained according to agreed protocols [1] and the general quality and comparability of the data are evaluated through an annual external quality assessment exercise distributed to the participating laboratories.

Data on *E. coli*, *K. pneumoniae*, *P. aeruginosa*, and *Acinetobacter* spp. isolates reported to EARS-Net for the year 2016 were extracted from The European Surveillance System (TESSy) database at ECDC. Data included isolates from blood and cerebrospinal fluid, both considered as markers of bloodstream infections. Data were de-duplicated to only include the first isolate per species, patient and year. For this study, only data from laboratories reporting observations for at least three of the four above-mentioned species were included. The study was limited to only include antibiotics commonly used for first-line treatment of bacteremia caused by Gram-negative species and routinely included in susceptibility testing in most local clinical laboratories in Europe. The AST information for the following antibiotic–species combinations were included in the study dataset: third-generation cephalosporins (ceftriaxone, ceftazidime or cefotaxime for *E. coli*, and *K. pneumoniae*; ceftazidime for *P. aeruginosa*), carbapenems (meropenem or imipenem, for all four species) and fluoroquinolones (ciprofloxacin, levofloxacin or ofloxacin for *E. coli* and *K. pneumoniae*; ciprofloxacin or levofloxacin for *P. aeruginosa* and *Acinetobacter* spp.) Isolates were considered as non-susceptible when reported as either intermediately susceptible (I) or resistant (R).

Of the 829 laboratories that reported data on any of the targeted species to EARS-Net for the year 2016 (total: 176,082 isolates), 749 fulfilled the inclusion criterion (total: 173,540 isolates) and were included in the final analysis.

### Statistical analysis

The percentages of *E. coli*, *K. pneumoniae*, *P. aeruginosa*, and *Acinetobacter* spp. isolates among the total of isolates included in the study, as well as the sum of the percentages of *P. aeruginosa* and *Acinetobacter* spp. (PSEACI) were calculated for each country. Rank comparison of the percentages of acquired non-susceptibility in each of the four Gram-negative species with the percentage of PSEACI was performed using the Spearman’s rank correlation coefficient to assess the monotonic relationships and limit impact of outliers. 

All statistical analyses were performed using Stata Statistical Software Release 14 (StataCorp, College Station, United States).

### Ethical statement

Antimicrobial resistance is listed as a special health issue in the EU case definitions for which ECDC routinely collects, analyses and disseminates surveillance data as stated by the Article 3 of its founding regulation. TESSy data are pseudonymised and processed for public interest in the area of public health. Approval of the study by an ethics committee was therefore not necessary.

## Results

### Distribution of the species

Overall, *E. coli* was the most commonly reported species (70.5%), followed by *K. pneumoniae* (17.4%), *P. aeruginosa* (8.9%) and *Acinetobacter* spp. (3.2%) (Table 1). The species distribution varied considerably between countries. Although *E. coli* remained the most commonly reported species in all 30 countries, the percentage of *E. coli* ranged from 31.9% (Greece) to 81.0% (Iceland), with a median of 69.0%. For *K. pneumoniae*, the percentage ranged from 10.5% (Iceland) to 34.3% (Romania), with a median of 17.5%. For *P. aeruginosa*, the percentage ranged from 4.1% (Latvia) to 20.2% (Cyprus) (median 7.5%) and for *Acinetobacter* spp. from 0.5% (Finland) to 22.1% (Greece) (median 1.9%). The combined percentage of the two PSEACI species among the total number of isolates ranged from 5.5% (Norway) to 39.2% (Greece), with a median of 10.1%.

**Table 1:** Number of reported isolates (n = 176,082) and included isolates (n = 173,540) of the four targeted species, and percentage of total per country and species, EU/EEA, 2016

Country	Isolates reported to EARS-Net	Isolates included in this study^a^	*Escherichia coli*		*Klebsiella pneumoniae*		*Pseudomonas aeruginosa*		*Acinetobacter* species	
	N	n	n	%	n	%	n	%	n	%
Austria	7,310	7,300	5,276	72.3	1,247	17.1	696	9.5	81	1.1
Belgium	4,970	4,610	3,538	76.7	669	14.5	334	7.2	69	1.5
Bulgaria	564	479	190	39.7	146	30.5	56	11.7	87	18.2
Croatia	1,810	1,771	1,020	57.6	312	17.6	259	14.6	180	10.2
Cyprus	317	317	149	47.0	75	23.7	64	20.2	29	9.1
Czech Republic	4,982	4,818	3,026	62.8	1,304	27.1	431	8.9	57	1.2
Denmark	6,535	6,535	4,847	74.2	1,156	17.7	460	7.0	72	1.1
Estonia	949	905	667	73.7	174	19.2	56	6.2	8	0.9
Finland	5,983	5,983	4,833	80.8	770	12.9	352	5.9	28	0.5
France	16,387	16,387	11,337	69.2	2,608	15.9	1,988	12.1	454	2.8
Germany	20,359	20,186	15,619	77.4	2,809	13.9	1,320	6.5	438	2.2
Greece	4,097	4,095	1,305	31.9	1,183	28.9	704	17.2	903	22.1
Hungary	3,859	3,840	1,990	51.8	720	18.8	731	19.0	399	10.4
Iceland	237	237	192	81.0	25	10.5	17	7.2	3	1.3
Ireland	3,755	3,597	2,855	79.4	439	12.2	240	6.7	63	1.8
Italy	10,339	9,703	5617	57.9	2,191	22.6	1,207	12.4	688	7.1
Latvia	446	393	218	55.5	85	21.6	16	4.1	74	18.8
Lithuania	1,284	1,265	783	61.9	321	25.4	74	5.8	87	6.9
Luxembourg	545	545	419	76.9	78	14.3	40	7.3	8	1.5
Malta	477	477	328	68.8	102	21.4	40	8.4	7	1.5
Netherlands	8,184	7,841	6,123	78.1	1,067	13.6	543	6.9	108	1.4
Norway	4,689	4,689	3,618	77.2	811	17.3	227	4.8	33	0.7
Poland	4,674	4,557	2,641	58.0	1,128	24.8	403	8.8	385	8.4
Portugal	9,575	9,513	5,740	60.3	2,338	24.6	1,229	12.9	206	2.2
Romania	1,017	956	403	42.2	328	34.3	82	8.6	143	15.0
Slovakia	1,601	1,570	807	51.4	458	29.2	191	12.2	114	7.3
Slovenia	1,890	1,890	1,420	75.1	267	14.1	143	7.6	60	3.2
Spain	9,429	9,389	6,761	72.0	1,679	17.9	843	9.0	106	1.1
Sweden	9,066	8,975	6,921	77.1	1,495	16.7	473	5.3	86	1.0
United Kingdom	30,752	30,717	23,685	77.1	4,232	13.8	2,186	7.1	614	2.0
**Total**	**176,082**	**173,540**	**122,328**	**70.5**	**30,217**	**17.4**	**15,405**	**8.9**	**5,590**	**3.2**

EARS-Net: European Antimicrobial Resistance Surveillance Network; EU/EEA: European Union and European Economic Area.

^a^ Limited to laboratories reporting observations for at least three of the four species *E. coli*, *K. pneumoniae*, *P. aeruginosa and Acinetobacter* spp.

### Antibiotic non-susceptibility

#### Third-generation cephalosporins

The percentage of isolates with acquired non-susceptibility to third-generation cephalosporins ranged from 4.7% (Iceland) to 41.0% (Bulgaria) in *E. coli* (median: 14.4%), from 0.0% (Iceland) to 75.9% (Bulgaria) in *K. pneumoniae* (median: 31.1%) and from 0.0% (Iceland) to 48.1% (Romania) in *P. aeruginosa* (median: 12.5%) (Table 2).

**Table 2:** Percentage of non-susceptible isolates (I or R) per country, antibiotic group and species, EU/EEA, 2016 (n = 173,540)

Country	*Escherichia coli*			*Klebsiella pneumoniae*			*Pseudomonas aeruginosa*			*Acinetobacter* species		Composite % IR to broad-spectrum β-lactams^a^	Composite % IR to FQ^b^
	% IR to 3GC	% IR to car	% IR to FQ	% IR to 3GC	% IR to car	% IR to FQ	% IR to 3GC	% IR to car	% IR to FQ	% IR to car	% IR to FQ		
Austria	10.4	<0.1	20.5	10.6	0.9	11.8	11.6	17.0	9.1	13.6	16.0	11.1	17.9
Belgium	11.1	0.1	25.4	23.5	3.0	27.1	8.3	12.6	17.1	1.5	11.8	12.9	24.8
Bulgaria	41.0	1.1	43.3	75.9	6.9	64.8	38.9	33.9	35.7	77.0	64.4	57.4	52.8
Croatia	15.6	0.0	29.4	50.0	1.9	46.3	20.5	47.9	39.9	95.0	94.8	34.4	40.4
Cyprus	30.2	0.7	47.0	32.0	12.0	37.3	10.9	26.6	21.9	71.4	71.4	33.5	41.8
Czech Republic	16.2	0.1	31.6	52.7	0.4	50.7	18.6	20.5	33.0	7.0	17.5	26.4	36.7
Denmark	8.1	0.0	13.8	9.9	0.4	8.3	4.5	4.6	5.0	0.0	2.8	8.1	12.1
Estonia	10.5	0.0	14.9	34.5	0.6	35.6	17.6	22.2	5.4	37.5	40.0	16.1	18.5
Finland	7.6	<0.1	12.3	5.3	0.3	4.9	5.4	10.8	9.6	0.0	0.0	7.5	11.1
France	12.1	<0.1	19.4	30.1	0.9	31.5	11.7	19.2	16.4	7.8	15.7	15.7	20.9
Germany	11.8	<0.1	20.6	14.3	0.7	14.9	11.0	18.0	18.8	5.4	8.5	12.4	19.4
Greece	19.0	1.5	32.5	73.2	67.1	70.4	38.6	75.1	38.5	95.6	95.9	60.8	58.1
Hungary	16.9	0.0	27.2	37.6	1.0	36.2	20.7	36.8	25.6	61.2	67.8	29.1	32.8
Iceland	4.7	0.0	10.1	0.0	0.0	0.0	0.0	5.9	23.5	0.0	0.0	4.2	10.0
Ireland	12.4	<0.1	24.1	15.5	0.9	15.7	12.5	12.1	15.0	0.0	3.2	12.5	22.1
Italy	31.2	0.4	45.5	59.3	37.2	60.4	23.0	28.3	30.1	79.9	80.6	40.7	49.5
Latvia	25.7	0.0	29.6	45.9	5.9	48.2	26.7	37.5	43.8	75.7	86.7	39.9	43.5
Lithuania	15.1	0.0	20.0	57.3	0.6	55.8	10.8	21.6	16.4	85.1	87.4	31.0	33.5
Luxembourg	13.6	0.0	29.2	35.9	1.3	42.3	10.0	19.4	22.5	0.0	25.0	17.0	30.5
Malta	14.9	0.3	42.4	22.5	8.8	37.3	12.5	12.5	15.0	42.9	42.9	16.8	39.0
Netherlands	7.0	0.0	14.0	9.8	0.1	10.2	3.5	6.1	9.0	1.9	4.7	7.3	13.1
Norway	6.1	0.1	11.9	7.4	0.0	6.1	7.1	11.6	8.4	0.0	3.0	6.6	10.6
Poland	15.2	<0.1	37.2	65.4	3.9	68.1	19.5	31.5	33.3	69.6	83.1	33.7	48.5
Portugal	16.8	0.1	30.2	48.6	6.4	48.8	19.9	22.4	23.4	52.2	51.2	26.1	34.4
Romania	22.7	0.8	30.4	68.9	38.7	64.7	48.1	54.9	52.4	85.3	90.9	50.7	53.2
Slovakia	31.2	0.0	42.0	62.4	3.3	68.1	31.1	46.2	48.4	32.4	46.5	42.2	50.8
Slovenia	13.8	0.4	25.8	25.1	0.4	34.5	17.5	23.8	22.4	45.0	55.0	17.1	27.7
Spain	15.4	0.1	33.4	23.0	3.8	24.8	15.6	24.7	27.5	64.2	68.9	18.1	31.8
Sweden	8.8	0.2	14.6	6.0	0.4	7.0	7.4	13.6	7.0	2.4	4.7	8.5	12.8
United Kingdom	10.0	0.1	17.1	9.9	0.6	9.5	5.3	7.3	9.8	2.6	4.8	9.6	15.3

3GC: third-generation cephalosporins; car: carbapenems; EU/EEA: European Union/European Economic Area; FQ: fluoroquinolones; IR: non-susceptible isolates.

^a^ Composite percentage of *E. coli* non-susceptible to third-generation cephalosporins, *K. pneumoniae* non-susceptible to third-generation cephalosporins, *P. aeruginosa* non-susceptible to carbapenems and *Acinetobacter* spp. non-susceptible to carbapenems among all tested *E. coli*, *K. pneumoniae*, *P. aeruginosa* and *Acinetobacter* spp. isolates.

^b^ Composite percentage of *E. coli* non-susceptible to fluoroquinolones, *K. pneumoniae* non-susceptible to fluoroquinolones, *P. aeruginosa* non-susceptible to fluoroquinolones and *Acinetobacter* spp. non-susceptible to fluoroquinolones among all tested *E. coli*, *K. pneumoniae*, *P. aeruginosa* and *Acinetobacter* spp. isolates.

#### Carbapenems

The percentage of isolates with acquired non-susceptibility to carbapenems ranged from 0.0% (Croatia, Denmark, Estonia, Hungary, Iceland, Latvia, Lithuania, Luxembourg, the Netherlands and Slovakia) to 1.5% (Greece) in *E. coli* (median: < 0.1%), from 0.0% (Iceland and Norway) to 67.1% (Greece) in *K. pneumoniae* (median: 1.0%), from 4.6% (Denmark) to 75.1% (Greece) in *P. aeruginosa* (median: 21.0%) and from 0.0% (Denmark, Finland, Iceland, Ireland, Luxembourg and Norway) to 95.6% (Greece) in *Acinetobacter* spp. (median: 35.0%) (Table 2).

#### Fluoroquinolones

The percentage of isolates with acquired non-susceptibility to fluoroquinolones ranged from 10.1% (Iceland) to 47.0% (Cyprus) in *E. coli* (median: 26.5%), from 0% (Iceland) to 70.4% (Greece) for *K. pneumoniae* (median: 35.9%), from 5.0% (Denmark) to 52.4% (Romania) in *P. aeruginosa* (median: 22.1%), and from 0% (Finland and Iceland) to 95.9% (Greece) in *Acinetobacter* spp. (median: 41.4%) (Table 2).

### Combining the percentages of susceptible (S) and non-susceptible (I + R) isolates in each of the four Gram-negative species

**Figure 1:**
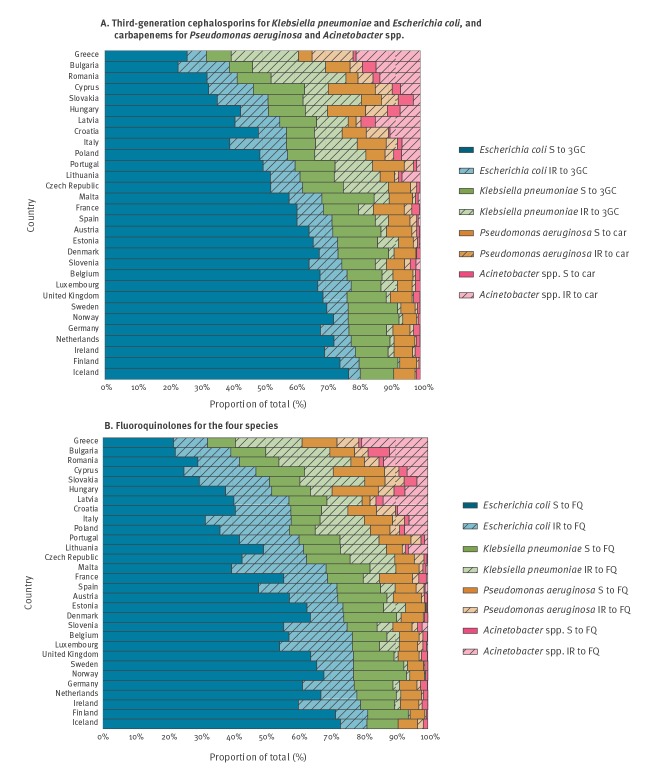
Distribution of fully susceptible (S) and non-susceptible (I or R) isolates in four Gram-negative species isolated from blood or cerebrospinal fluid, 30 EU/EEA countries, 2016 (n = 173,540)

Figure 1 shows, within each of the four Gram-negative species reported by the 30 countries, the distribution of isolates fully susceptible (S) and non-susceptible (I + R) to broad-spectrum β-lactams (third-generation cephalosporins for *K. pneumonia*e and *E. coli*, carbapenems for *P. aeruginosa* and *Acinetobacter* spp.) (Figure 1A) and to fluoroquinolones for the four species (Figure 1B). The sum of these non-susceptible isolates, expressed as the composite percentage of isolates intermediately susceptible and resistant to broad-spectrum β-lactams, ranged from 4.2% for Iceland and 6.6% for Norway to 15.7% for France and 18.1% for Spain, up to 57.4% for Bulgaria and 60.8% for Greece (Table 2). In Figure 1B, the composite percentage of isolates intermediately susceptible and resistant to fluoroquinolones ranged from 10.0% for Iceland and 10.6% for Norway to 20.9% for France and 31.8% for Spain, up to 52.8% for Bulgaria and 58.1% for Greece. Among the total of isolates from bloodstream infection involving the four Gram-negative species, the proportion of *E. coli* isolates susceptible to third-generation cephalosporins ranged from 77.5% in Iceland to only 24.3% in Bulgaria, whereas the proportion of *Acinetobacter* spp. isolates non-susceptible to carbapenems ranged from 0% in Denmark, Finland, Iceland, Ireland, Luxembourg and Norway to 21.7% in Greece.

3GC: third-generation cephalosporins; car: carbapenems; EU/EEA: European Union/European Economic Area; FQ: fluoroquinolones; I: intermediately susceptible; R: resistant; S: susceptible.

The countries are ranked by increasing proportion of *Escherichia coli* isolates. Colour shading: susceptible isolates; hatched areas: non-susceptible isolates.

### Correlation between country-specific percentages of acquired non-susceptibility in the four species and proportion of *Pseudomonas aeruginosa* and *Acinetobacter* spp. 

**Figure 2:**
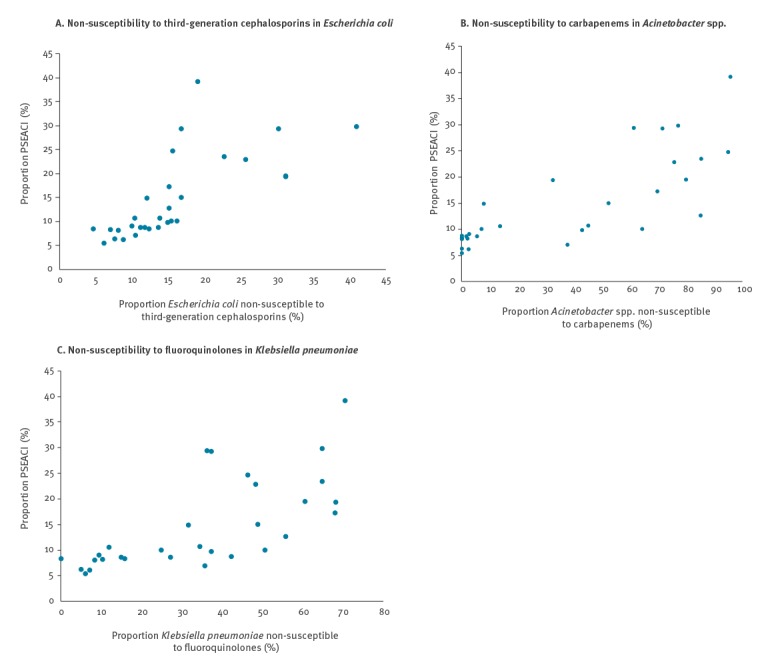
Scattergrams showing the sum of proportions of *Pseudomonas aeruginosa* and *Acinetobacter* spp. combined (PSEACI) (n = 20,995) among four Gram-negative species^a^ and proportions of various acquired non-susceptibility and species, 30 EU/EEA countries, 2016

There was a strong positive correlation between the ranks of the country-specific percentages of non-susceptibility to third-generation cephalosporins and the percentage of PSEACI: *E. coli* (rho = 0.88, p < 0.0001) (Figure 2A), *K. pneumoniae* (rho = 0.82, p < 0.0001) and *P. aeruginosa* (rho = 0.78, p < 0.0001). Similar positive correlations were found for non-susceptibility to carbapenems in *K. pneumoniae* (rho = 0.76, p < 0.0001), *P. aeruginosa* (rho = 0.85, p < 0.0001) and *Acinetobacter* spp. (rho 0.85, p < 0.0001). The correlation was more moderate and not statistically significant for non-susceptibility to carbapenems in *E. coli* (r = 0.33, p = 0.077) (Figure 2B). Finally, this correlation was also strong for non-susceptibility to fluoroquinolones in *E. coli* (rho = 0.75, p < 0.0001) *K. pneumoniae* (rho = 079, p < 0.0001), P. aeruginosa (rho = 0.79, p < 0.0001) and *Acinetobacter* spp. (rho = 0.85, p < 0.0001) (Figure 2C).

Each dot represents a country.

^a^ *Escherichia coli*, *Klebsiella pneumoniae*, *Pseudomonas aeruginosa* and *Acinetobacter* spp.

## Discussion

The intrinsic resistance profiles of different bacteria are reflected in the European Committee on Antimicrobial Susceptibility Testing (EUCAST) expert rules [5] as well as in the number of antimicrobial drugs that must be considered to define acquired MDR/XDR/PDR patterns [6]. As a consequence, for Gram-negative bacteria, identification of the species isolated from a positive blood culture provides immediate information to the microbiologist and the clinician about which antimicrobials should not be used because of intrinsic resistance in that species. This is one of the major reasons for the development of rapid methods for bacterial species identification, such as matrix-assisted laser desorption/ionization time-of-flight (MALDI-TOF) mass-spectrometry. In addition, acquired resistance is recognised as a major global public health issue [11,12] because it jeopardises the effectiveness of antimicrobial drugs that are normally active against intrinsically multi-susceptible species such as *E. coli* and because it further reduces the possibility to treat infections with species that are already intrinsically resistant to many antimicrobial drugs such as *P. aeruginosa* and *Acinetobacter* spp.

The results presented here, based on an analysis of EARS-Net data for the year 2016 and focusing on four major Gram-negative species (*E. coli*, *K. pneumoniae*, *P. aeruginosa* and *Acinetobacter* spp.) isolated from bloodstream infections, clearly show significant statistical association between the distribution of species, and the percentages of acquired non-susceptibility to major antibiotic groups. All but one correlation were statistically significant, with Spearman’s rank correlation coefficient being > 0.75 for 10 of the 11 pairs of variables tested, which is generally considered as indicating a strong correlation [13,14]. In short, the higher the proportion of the two most intrinsically resistant species *P. aeruginosa* and *Acinetobacter* spp., the higher the percentages of acquired non-susceptibility in *E. coli*, *K. pneumoniae*, *P. aeruginosa* and *Acinetobacter* spp., a point that directly indicates the burden of intrinsic resistance. Consequently, there were, at one extremity of the EU/EEA gradient, countries with a very low proportion of bloodstream infections caused by the two most intrinsically resistant species together with low percentages of isolates with acquired non-susceptibility in any of the four Gram-negative species. At the opposite extremity of the EU/EEA gradient, countries with high percentages of bloodstream isolates caused by the two most intrinsically resistant species had high percentages of isolates with acquired non-susceptibility in all the four species. The weak statistical link noted for carbapenem non-susceptibility in *E. coli* could be due to very low percentages of non-susceptibility reported from a majority of the countries [1].

Statistical association is not equal to causation and the correlations presented in this study do not mean that there is a cause-and-effect relationship between intrinsic and acquired resistance. However, we can hypothesise that the two major driving forces of antibiotic resistance, i.e. the use of antibiotics acting as a selective pressure on resistant bacteria and the spread of the selected antibiotic-resistant bacteria by cross-transmission between humans, animals and the environment, apply to both the intrinsically resistant species such as *P. aeruginosa* and *Acinetobacter* spp. (and to a lesser extent *K. pneumoniae*), and to strains with acquired resistance (in the present study: resistance to β-lactams or fluoroquinolones). Antibiotic use has a strong selective effect on *P. aeruginosa* and *Acinetobacter* spp [15,16] and on strains with acquired resistance traits in many species [17-20]. Host-to-host cross-transmission of *P. aeruginosa* and *Acinetobacter* spp [21,22] and of strains with acquired resistance traits e.g. in enterobacteria [23,24] also plays a crucial role in the spread of resistance. Indeed, the available comparative data suggest that the EU/EEA countries that in our study had the highest proportion of intrinsically resistant species and percentages of isolates with acquired resistance, generally also have the highest antibiotic consumption in humans (in particular broad-spectrum β-lactams and fluoroquinolones) and the lowest levels of preventive measures against cross-transmission of microorganisms in hospitals such as consumption of alcohol hand rub solutions, proportion of rooms with a single bed and staffing of infection control teams [2,25,26]. Differences in healthcare systems or, possibly, climate issues could also be involved in the discrepancies between countries.

This study has several limitations. Firstly, although a very large number of isolates from bloodstream infections (n = 173,540) were analysed, this study only included the four Gram-negative species covered by EARS-Net (*E. coli, K. pneumoniae, P. aeruginosa* and *Acinetobacter* spp.) and did not cover other species such as* Enterobacter cloacae, Serratia marcescens* or *Proteus mirabilis.* However, the included four species taken together constitute the majority of invasive aerobic Gram-negative isolates in many studies: 78% of bloodstream infections recorded by the SENTRY surveillance programme organised in Europe from 1997 to 1998 [27], 73% in the European point prevalence survey coordinated by ECDC in 2011 and 2012 [2] and 74% in a meta-analysis on infections recorded in developing countries [28]. Secondly, our study did not cover all antibiotics but only a selection of broad-spectrum β-lactams (third-generation cephalosporins and carbapenems) and the fluoroquinolones, widely used for treating bacteraemia caused Gram-negative species. However, we observed in the same data source similar types of correlations with aminoglycosides, another major class of antibiotics used for treating such severe infections (data not shown). Finally, the patient case-mix, which depends on the types of included hospitals and on the frequency of blood culture sampling in each country, might have had an impact on the reported resistance percentages. Importantly, in the EARS-Net reports that provide detailed information on the number of laboratories and characteristics of the hospitals included, there were no marked differences between countries with low and high resistance percentages concerning the proportions of tertiary care hospital beds and intensive care unit beds, two types of hospital settings where resistance rates are usually the highest [1,29]. In addition, the representativeness of the population sample for 2016 data has been assessed as high in 23 of the 30 countries [29]. However, there was a trend towards a lower number of blood culture sets taken per 1,000 patient days in some of the countries with the highest percentages of resistance [29], which may have led us to overestimate the percentage of acquired resistance or the percentage of PSEACI in these countries. Concerning the quality of antibiotic susceptibility testing in EARS-Net, the widespread implementation of EUCAST clinical breakpoints in Europe and the high proportion of laboratories that participated in 2016 in the annual EARS-Net external quality assessment exercises with satisfactory results [1] greatly helps to ascertain the ability of the EU/EEA countries to report robust and trustworthy antimicrobial resistance data to EARS-Net.

## Conclusion

We observed a strong correlation in bloodstream infections between on the one hand the countries with most intrinsically resistant Gram-negative species, indicating the burden of intrinsic resistance, and on the other hand the percentage of acquired non-susceptibility in these species. This important information adds to the already well-established arguments for a strong reduction in the consumption of antibiotics, particularly those with broad-spectrum activity, which exert a selective pressure on all types of resistant bacteria. It also reinforces the crucial importance of measures to prevent host-to-host cross-transmission of antibiotic-resistant microorganisms, not only to control acquired resistance in every bacterial species but also to limit the burden of infections caused by species such as *P. aeruginosa* and *Acinetobacter* spp*.,* in which intrinsic resistance per se represents a therapeutic problem.

## EARS-Net participants group

**Austria:** Reinhild STRAUSS,  Federal Ministry for Labour, Social Affairs, Health and Consumer Protection; Vienna, Austria; **Belgium**: Karl MERTENS, Sciensano, Brussels, Belgium; **Bulgaria:** Yuliya Stoyanova MARTEVA-PROEVSKA, Central Laboratory of Clinical Microbiology, University Multiprofile Hospital for Active Treatment (UMHAT), Bulgaria; **Croatia:** Silvija ŠOPREK, University Clinic for Infectious Diseases “Dr. Fran Mihaljević”, Zagreb, Department for Clinical Microbiology, Zagreb, Croatia; **Cyprus:** Panagiota MAIKANTI-CHARALAMPOUS, Microbiology Department- National Reference Laboratory for Antimicrobial Resistance Surveillance, Nicosia General Hospital 215, Nicosia, CYPRUS; **Czech Republic**: Vladislav JAKUBU, National Institute of Public Health, Prague, Czech Republic; **Denmark**: Ute SÖNKSEN, Statens Serum Institut, Copenhagen, Denmark; **Estonia:** Marina IVANOVA, East Tallinn Central Hospital Central Laboratory, Tallinn, Estonia; **France:** Sylvie MAUGAT, Santé Publique France, the French Public Health Agency, Saint-Maurice, France; **Finland:** Jari JALAVA, Infectious Disease Control and Vaccinations Unit, National Institute for Health and Welfare, Helsinki, Finland; **Germany:** Ines NOLL, Healthcare-associated infections, surveillance of antimicrobial resistance and consumption Department for Infectious Disease Epidemiology Robert Koch Institute, Berlin, Germany ; **Greece:** Michalis POLEMIS, Hellenic National Public Health Organization , Athens, Greece ; **Hungary:** Zsolt VEGH, Directorate of Clinical and Public Health Microbiology, National Public Health Institute, Budapest, Hungary ; **Iceland**: Karl G. KRISTINSSON, Clinical Microbiology, Landspitali University Hospital, Reykjavik, Iceland ; **Ireland:** Stephen MURCHAN, Health Protection Surveillance Centre, Dublin 1, Ireland; **Latvia**: Arta Olga BALODE, Department of Biology and Microbiology, Rīga Stradiņš University, Riga, Latvia; **Lithuania:** Jolanta MICIULEVICIENE , National Public Health Surveillance Laboratory, Vilnius, Lithuania ; **Luxembourg**: Monique PERRIN, Laboratoire National de Santé, Dudelange, Luxembourg; **the Netherlands:** Sjoukje H. S. WOUDT, Centre for Infectious Disease Control (CIb), National Institute for Public Health and the Environment (RIVM), Bilthoven, the Netherlands; **Norway:** Frode W GRAN, St. Olav University Hospital, Trondheim, Norway; **Poland:** Waleria HRYNIEWICZ, National Medicines Institute, Warsaw, Poland; **Portugal:** Manuela CANIÇA, National Institute of Health Doutor Ricardo Jorge, Lisboa, Portugal; **Romania**: Andreea Sorina NICULCEA, National Institute of Public Health, Bucharest, Romania; **Slovakia: **Eva SCHRETEROVA**,** Louis Pasteur University Hospital Kosice, Kosice, Slovakia; **Slovenia:** Maja**Š**UBELJ****, National Institute of Public Health, Ljubljana, Slovenia; **Spain:** Belén ARACIL, Reference and Research Laboratory on Antimicrobial Resistance, Centro Nacional de Microbiología, Instituto de Salud Carlos III, Madrid, Spain; **Sweden:** Hanna BILLSTRÖM, Public Health Agency of Sweden, Solna, Sweden and The Swedish Antimicrobial Resistance Surveillance Network (Svebar); **United Kingdom**: Eleanor ANDERSON, Health Protection Scotland - NHS National Services Scotland.
